# Neuroimaging Recommendations in Outpatients With Dementia: Three Cases of Frontal Meningioma Demonstrating Reversible Dementia

**DOI:** 10.7759/cureus.14028

**Published:** 2021-03-21

**Authors:** Satoshi Matsuo, Toshiyuki Amano, Yuichiro Miyamatsu, Yutaka Fujioka, Akira Nakamizo

**Affiliations:** 1 Neurological Surgery, Kyushu Medical Center, Fukuoka, JPN

**Keywords:** benign brain tumor, cognitive impairment, frontal meningioma, reversible dementia

## Abstract

Benign brain tumors largely affect the brain and can lead to reversible dementia, which can be resolved following the treatment of the primary etiology. Herein, we report three cases of relatively large frontal meningiomas in patients who presented with cognitive impairment as initial symptoms. The three participants demonstrated notable dementia alongside frontal meningioma. Following resection, all patients showed dramatic cognitive function improvement, and they successfully returned to society. Our cases illustrate the benefit of active surveillance with neuroimaging in selected patients, especially those who present with acute or subacute dementia.

## Introduction

Reversible dementia is a form of dementia that is resolved following the treatment of the primary etiology. The early detection of hidden treatable causes is important for physicians treating reversible dementia.

Brain tumors can cause dementia due to the mass effect on the adjacent brain tissue. Meningioma, a benign brain tumor, has been found to cause reversible dementia if it is located in the frontal region of the brain [1-3]. Meningioma diagnosis can be delayed when patients manifest only psychiatric symptoms [1,4]. Generally, impaired cognitive function is significantly improved after surgical removal [5]. Therefore, early diagnosis and surgical treatment are critical; however, most general physicians are not familiar with the importance of neuroradiological examination for patients who present with dementia. Herein, we report three cases of relatively large frontal meningiomas that presented with reversible dementia.

## Case presentation

Case 1

**Figure 1:**
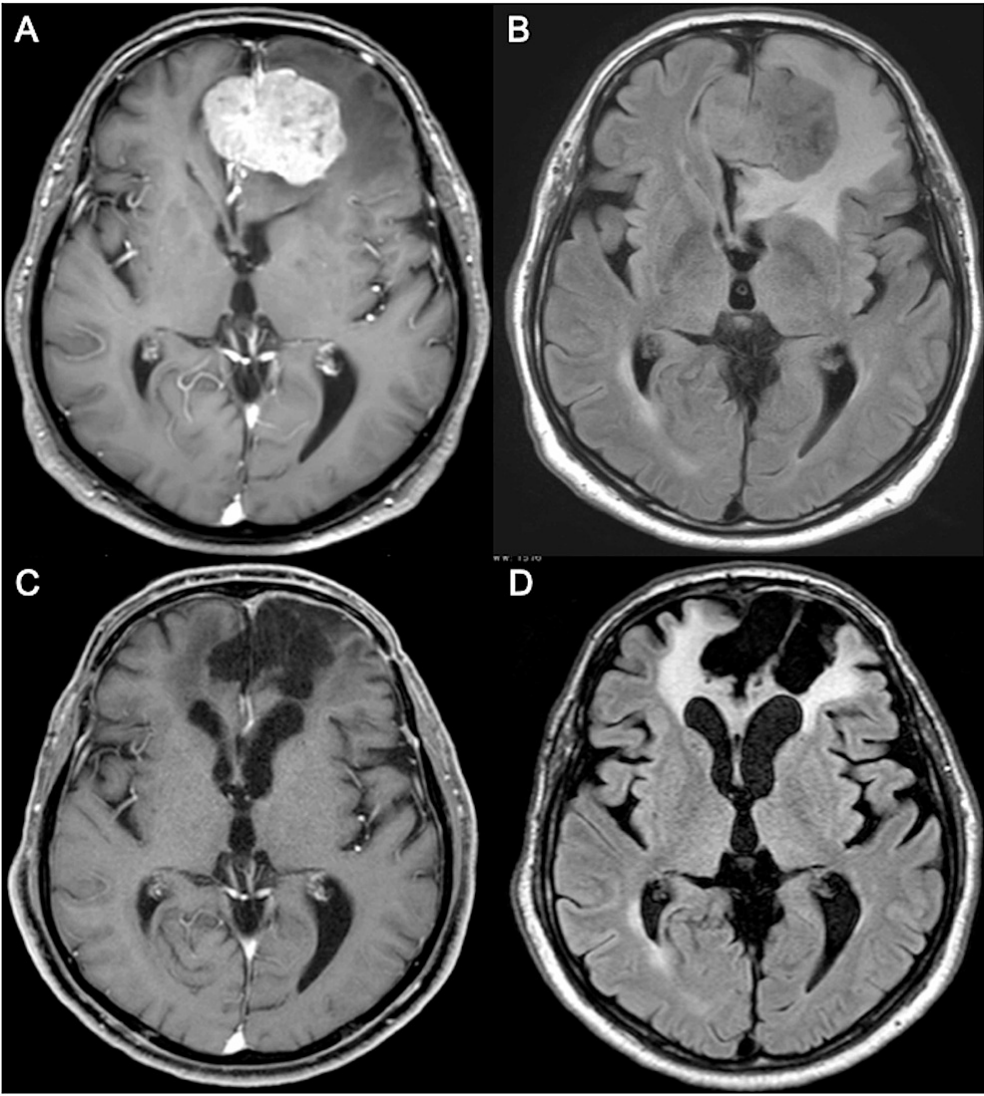
Pre- and postoperative magnetic resonance image of Case 1. (A) Preoperative Gd enhanced axial T1WI. (B) Preoperative axial FLAIR image. (C) Postoperative Gd enhanced axial T1WI. (D) Postoperative axial FLAIR image. Gd: gadolinium; T1WI: T1-weighted image; FLAIR: fluid-attenuated inversion recovery

A 79-year-old man with a history of hyperuricemia and hypertension developed incontinence and apathy two days before admission and presented to his primary care physician. His head CT images showed an approximately 5 cm mass lesion in the frontal region, and he was transferred to our department. On admission, his cognitive examination results were as follows, suggesting cognitive impairment: Japan Coma Scale (JCS) I-1, Glasgow Coma Scale (GCS) E4V4M6, Mini-Mental State Examination (MMSE) 25/30, Hasegawa Dementia Rating Scale-Revised (HDS-R) 22/30, Frontal Assessment Battery (FAB) 8/18, and Trail Making Test (TMT; Part A 200 s, Part B 634 s). Contrast-enhanced MRI revealed a homogeneously enhanced 4.8x4.0x4.0 cm mass with peritumoral edema at the midline of the frontal region, consistent with falx meningioma (Figures 1A, 1B). We performed surgical resection of the tumor and achieved gross total resection (Simpson grade 1; Figures 1C, 1D). Histological examination confirmed the meningioma condition (World Health Organization {WHO} grade 1). The patient’s postoperative course was uneventful, and he was transferred to the rehabilitation hospital for cognitive function training, 21 days after surgery. He returned to his ordinary life after being discharged from a rehabilitation hospital. His cognitive examinations at discharge (approximately 70 days after surgery) were as follows, suggesting improvement of the cognitive impairment: JCS 0, GCS E4V5M6, MMSE 27/30, HDS-R 24/30, FAB 10/18, and TMT (Part A 111 s, Part B 246 s). 

Case 2

**Figure 2:**
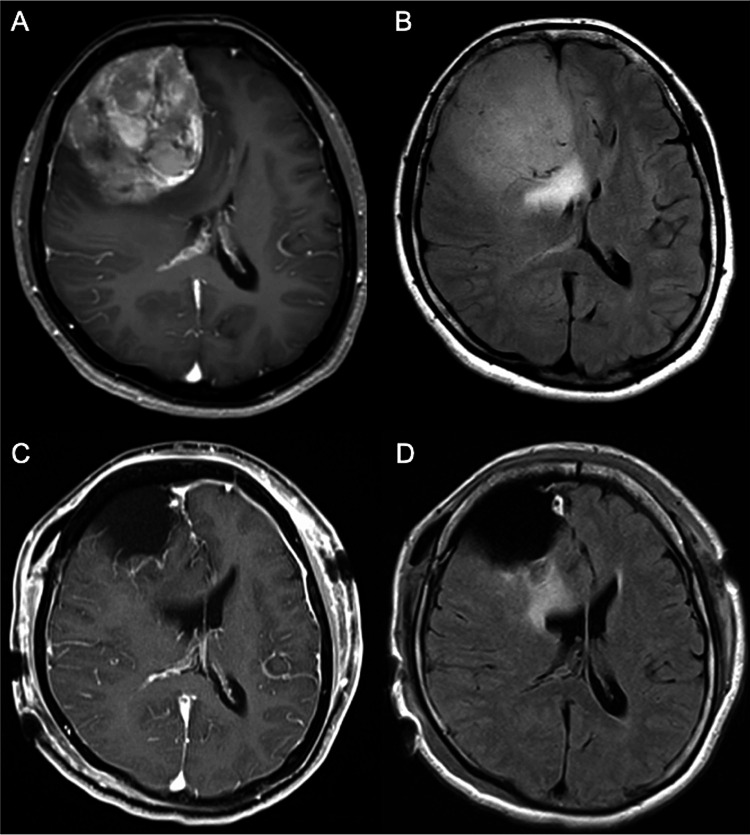
Pre- and postoperative magnetic resonance images of Case 2. (A) Preoperative Gd enhanced axial T1WI. (B) Preoperative axial FLAIR image. (C) Postoperative Gd enhanced axial T1WI. (D) Postoperative axial FLAIR image. Gd: gadolinium; T1WI: T1-weighted image; FLAIR: fluid-attenuated inversion recovery

Six months prior to visiting our department, a 42-year-old woman presented with memory disturbances, anorexia, weight loss, and apathy. Initially, she assumed that these symptoms were caused by the menopausal syndrome. Five months later, following the lack of symptom improvement, she visited a psychiatric clinic and started using antidepressants after being diagnosed with depression. She complained of headaches and visited a neurosurgical clinic. MRI revealed a 7 cm lesion in the right frontal region, and she was referred to our department. Upon admission, her cognitive examination results were as follows, suggesting severe cognitive impairment: JCS I-1, GCS E4V4M6, MMSE 7/30, HDS-R 3/30, FAB 6/18, and Behavioral Assessment of the Dysexecutive Syndrome (BADS) 4/24. Contrast-enhanced MRI revealed a heterogeneously enhanced extra-axial mass of approximately 7 cm in the frontal region with peritumoral edema, which is consistent with right frontal convexity meningioma (Figures 2A, 2B). Preoperative endovascular feeder embolization and surgical removal of the tumor were performed. Gross total resection was achieved (Simpson grade 1; Figures 2C, 2D). Histological examination confirmed a WHO grade 1 meningioma. Her postoperative course was uneventful, and she was transferred to a rehabilitation hospital for cognitive function training (17 days after surgery). Her postoperative cognitive examinations (approximately 20 days after surgery) were as follows, suggesting improvement of the cognitive impairment: MMSE 30/30, HDS-R 30/30, FAB 16/18, BADS 23/24, and TMT (Part A 30 s, Part B 42 s). After she was discharged from the rehabilitation hospital, she returned to her normal life without requiring antidepressants.

Case 3

**Figure 3:**
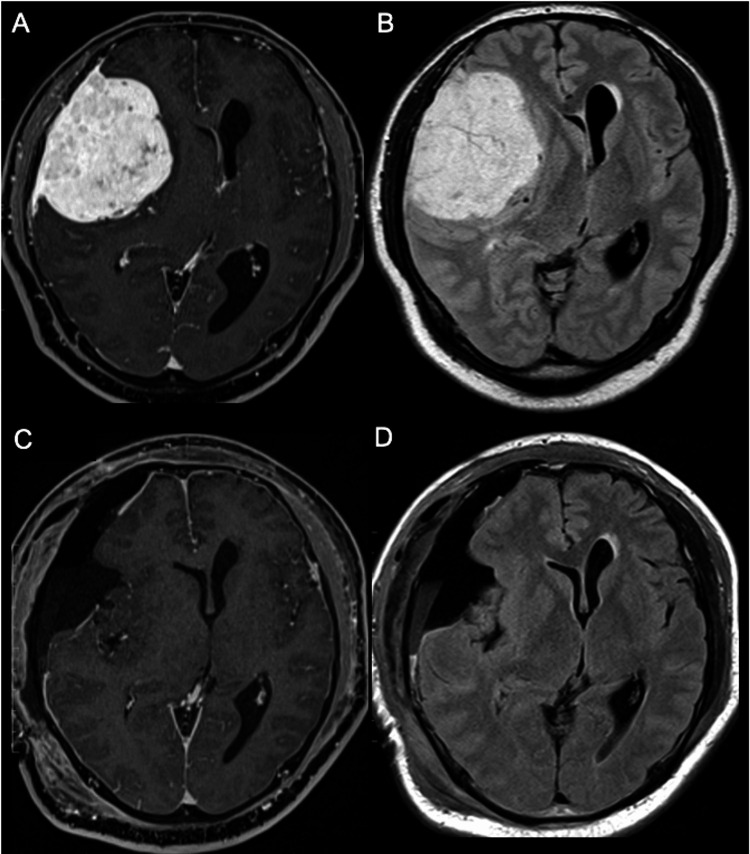
Pre- and postoperative magnetic resonance images of Case 3. (A) Preoperative Gd enhanced axial T1WI. (B) Preoperative axial FLAIR image. (C) Postoperative Gd enhanced axial T1WI. (D) Postoperative axial FLAIR image. Gd: gadolinium; T1WI: T1-weighted image; FLAIR: fluid-attenuated inversion recovery

Three months prior to admission, a 47-year-old man presented to a psychiatric clinic with apathy and restlessness. He started using antidepressants after being diagnosed with depression by psychiatrists. Later, he developed headaches and left hemiparesis and became ambulatory with a walking cane. He had a history of surgical procedures for lumbar canal stenosis seven years prior to admission, and his orthopedic surgeon had planned lumbar canal restenosis. Conversely, a head CT was performed by the doctor to address the headache complaints, which revealed a large mass in the right frontal region. The patient was then referred to our department. He had mild cognitive impairment and his neurological examination results on admission were: JCS I-1, GCS E4V4M6, mild lift hemiparesis (manual muscle testing: four of five), MMSE 25/30, HDS-R 26/30, FAB 13/18, and TMT (Part A 198 s, Part B unable). Contrast-enhanced MRI revealed an extra-axial approximately 7 cm homogeneously enhanced mass with a dural tail sign in the right frontal lobe (Figures 3A, 3B). We planned preoperative endovascular feeder embolization and surgical removal of the tumor. On the day when preoperative endovascular embolization was scheduled, he demonstrated anisocoria and rapidly progressive deterioration of consciousness. We performed emergency surgery and resected the tumor. Gross total resection was achieved (Simpson grade 1; Figures 3C, 3D). The postoperative course was uneventful, and his consciousness promptly recovered. Histological examination confirmed the meningioma condition (WHO grade 1). He became ambulatory and was discharged from the hospital 13 days after surgery and returned to his ordinary life, and the antidepressant usage was discontinued. His postoperative cognitive examinations (approximately a week after surgery) were as follows: JCS 0, GCS E4V5M6, MMSE 30/30, HDS-R 30/30, FAB 18/18, TMT (Part A 41 s, Part B 77 s), and Wechsler Adult Intelligence Scale-III (full IQ 101, verbal IQ 110, performance IQ 90).

## Discussion

We report three cases of relatively large meningiomas in the frontal region that initially manifested as psychiatric symptoms. All patients, including an elderly patient, showed dramatic recovery of cognitive function following tumor resection. 

Most meningiomas occur between the ages of 30 years and 70 years and rarely occur in children [3]. Frontal meningiomas commonly demonstrate psychiatric symptoms, including mood disorders, psychosis, memory issues, personality changes, anxiety, and anorexia nervosa [3,5,6]. As shown in Cases 1 and 2, being elderly or a menopausal woman can delay or prevent the search for the cause of cognitive dysfunction caused by frontal meningioma. 

In terms of age, approximately 15% of Japanese patients who were aged 65 years and older were affected by dementia in 2012 [7]. Thus, dementia is one of the most common diseases in elderly patients. Typically, dementia has an insidious onset and slow progression. As shown in Case 1, the acute and subacute onset of cognitive decline is generally regarded as a treatable cause [8]. Therefore, general physicians should investigate patients who present with acute or subacute dementia to determine whether there is a treatable cause. 

Moreover, middle-aged women are more than twice as likely as men to develop meningiomas [3]. As shown in Case 2, detecting the treatable cause may also be delayed by menopause in female patients as depression, anxiety, cognitive changes, and headache are common symptoms [9]. Middle-aged women and men are also likely to develop mood disorders due to work-related stress [10]. Therefore, as shown in Case 3, depression in middle-aged men is another possible situation that can delay surveillance for detecting a treatable cause. 

Large frontal meningiomas tend to affect the motor cortex, Broca’s area, and the large branch/main trunk of the anterior and middle cerebral arteries. Patients with large frontal meningiomas can depict not only psychiatric symptoms but also paralysis and aphasia. Thus, early diagnosis and surgical treatment are required. In Cases 2 and 3, headache, which is caused by elevated intracranial pressure, triggered neuroradiological examinations. Although headache is a common symptom in daily medical practice, the neuroradiological examination should be performed in patients who present with both psychiatric symptoms and headaches. 

According to the Japanese Ministry of Health, Labor and Welfare, in 2018, there were approximately 320,000 physicians, and approximately one-fifth (19.4%) were general physicians [11]. Approximately half of the physicians working at outpatient clinics in Japan specialize in internal medicine (51.0%), whereas those who specialize in neurology and neurosurgery are limited (2.2% and 1.4%, respectively) [11]. Therefore, general physicians seem to be likely to encounter patients with reversible dementia in daily medical practice. Given that the number of CT and MRI scanners per million population in Japan is the largest among the world (111.49 and 55.21 scanners/million, respectively) [12], Japanese general physicians are in an environment in which neuroradiological examinations are accessible. Although we should avoid the escalation of healthcare costs, neuroimaging examinations should be performed without hesitation to detect treatable causes in selected patients.

Surgical removal is the first-line treatment for symptomatic meningiomas. Even in elderly patients, surgical removal of the tumor is a safe and effective treatment option while preserving performance status [13]. In all our cases, frontal lobe function improved, and they returned to their ordinary life; however, several reports have suggested that patients with frontal meningioma who undergo surgical treatment can still demonstrate cognitive impairment in a wide range of cognitive functions [2,14]. This may be partly due to irreversible injury to the brain tissue due to tumor extension and/or surgical procedures. 

## Conclusions

General physicians should recognize that benign brain tumors can cause cognitive impairment and with proper treatment, remission is probable. In addition, neuroradiological examinations should not be hesitated, especially in patients with acute or subacute dementia to detect the treatable causes.
